# Bacterial Pore-Forming Toxins Promote the Activation of Caspases in Parallel to Necroptosis to Enhance Alarmin Release and Inflammation During Pneumonia

**DOI:** 10.1038/s41598-018-24210-8

**Published:** 2018-04-11

**Authors:** Norberto Gonzalez-Juarbe, Kelley M. Bradley, Ashleigh N. Riegler, Luis F. Reyes, Terry Brissac, Sang-Sang Park, Marcos I. Restrepo, Carlos J. Orihuela

**Affiliations:** 1 Department of Microbiology, The University of Alabama at Birmingham, 845 19th Street South, Birmingham, Alabama 35294-2170 USA; 2 Division of Pulmonology, South Texas Veterans Health Care System, San Antonio, Texas 78229 USA

## Abstract

Pore-forming toxins are the most common virulence factor in pathogenic bacteria. They lead to membrane permeabilization and cell death. Herein, we show that respiratory epithelial cells (REC) undergoing bacterial pore-forming toxin (PFT)-induced necroptosis simultaneously experienced caspase activation independently of RIPK3. MLKL deficient REC treated with a pan-caspase inhibitor were protected in an additive manner against PFT-induced death. Subsequently, cleaved versions of caspases-2, -4 and -10 were detected within REC undergoing necroptosis by immunoblots and monoclonal antibody staining. Caspase activation was observed in lung samples from mice and non-human primates experiencing Gram-negative and Gram-positive bacterial pneumonia, respectively. During apoptosis, caspase activation normally leads to cell shrinkage, nuclear condensation, and immunoquiescent death. In contrast, caspase activity during PFT-induced necroptosis increased the release of alarmins to the extracellular milieu. Caspase-mediated alarmin release was found sufficient to activate resting macrophages, leading to Interleukin-6 production. In a mouse model of Gram-negative pneumonia, deletion of caspases -2 and -11, the mouse orthologue of caspase-4, reduced pulmonary inflammation, immune cell infiltration and lung damage. Thus, our study describes a previously unrecognized role for caspase activation in parallel to necroptosis, and indicates that their activity plays a critical pro-inflammatory role during bacterial pneumonia.

## Introduction

According to the World Health Organization, pneumonia is the leading cause of infectious death worldwide^1^. Bacterial pathogens are at fault for approximately 150 million cases of pneumonia per year^2^. Pore-forming toxins (PFTs) have a nearly ubiquitous presence in bacterial pathogens and are often responsible for a large part of the tissue injury that occurs during infection^3–5^. To date, PFTs have been shown to cause apoptosis, necroptosis, and pyroptosis in a variety of *in vitro* and *in vivo* models^6^. Nonetheless, the molecular mechanisms of these ubiquitous toxins during disease and their effect on the host response continues to be elucidated today.

PFTs are virulence determinants by which bacteria mediate release of sequestered nutrients from host cells, kill or incapacitate immune cells to avoid clearance, or cause inflammation that promotes dispersal^7^. PFTs have been implicated in the activation of various modes of cell death. At high doses, PFTs mediate irreversible plasma membrane permeability leading to necrotic death, mainly due to loss of osmotic regulation and/or a metabolic catastrophe^8^. At sub lytic concentrations, PFTs have been shown to cause programmed cell death including apoptosis, pyroptosis, or necroptosis^5^. Apoptosis is an immunoquiescent cell death program regulated by cysteine-aspartic proteases, called caspases. Apoptotic caspase activation can be due to intrinsic signals, such as the release of cytochrome C from damaged mitochondria^9,10^, or extrinsic signals, including engagement of a death receptor on the cell membrane by its cognate ligand^11^. For example, *Helicobacter pylori* vacuolating toxin vacA can induce apoptosis through its permeabilization of the mitochondrial inner membrane, causing a metabolic breakdown in the cell and the release of cytochrome C, leading to the activation of caspase-9^12^. Alternatively, exposure to Fas ligand, triggers apoptosis through caspase-8 mediated extrinsic apoptosis^13^. Pertinent to this manuscript, apoptosis can also be initiated by less studied caspases such as caspase-2, the most evolutionarily conserved caspase, and caspase-10 upon contact with bacterial pathogens^11,14^. How this occurs is still unclear. PFTs have also been shown to induce programmed modes of necrotic death, specifically pyroptosis and necroptosis^7^. Pyroptosis is mediated by the activation of the canonical caspase-1 or the non-canonical caspase-4^15,16^. Caspase-4 and its murine orthologue, caspase-11, have been shown to mediate epithelial defenses against enteric bacterial pathogens by activating the inflammasome^16^. To date, the complete functions of caspases -2, -4, and 10 remain undefined^10,11,17,18^.

Necroptosis, a form of programmed necrosis, is highly inflammatory due to the purposeful release of cytosolic molecules containing danger-associate-molecular-patterns, i.e. alarmins. Necroptosis is modulated by receptor-interacting serine-threonine kinases -1 and -3 (RIPK1, RIPK3) and occurs when caspase-8, a pivotal apoptotic caspase, is inhibited. Briefly, caspase-8 inactivates both RIPK1 and RIPK3 by sequestration or proteolytic cleavage^19,20^. When cellular distress signals are present and caspase-8 is inactive, RIPK3 binds to RIPK1 forming the necroptosome. The necroptosome phosphorylates mixed lineage kinase domain-like protein (MLKL), which then integrates and damages cell membranes, leading to necrotic death^19–21^. Importantly, necroptosis is understood and is by definition exclusive of caspase activity^19,21^. This is supported by our own studies that showed no impact of caspase-inhibition on PFT-mediated necroptotic death of macrophages^22^. In addition, it has also been reported that caspase-8 activity suppresses RIPK3 activation^19^. Nonetheless, a recent report showed that during influenza A respiratory tract infection, caspase-8 induced apoptosis occurred in parallel to necroptosis in a RIPK3 dependent manner^23^. Thus, the distinction between apoptosis and necroptosis may not be so clear cut.

In recent work, we have shown that PFTs activate the necroptosis pathway in macrophages^22^ and respiratory epithelial cells (REC)^24^ and this contributes to pulmonary injury during bacterial pneumonia. PFT-induced necroptosis required RIPK1, RIPK3 and MLKL, yet was activated independently of death receptor engagement through non-canonical means, i.e. ion dysregulation as the result of membrane permeabilization^24^. One important distinction between macrophage and REC death is that only partial protection against PFTs was observed in REC following necroptosis inhibition. In contrast, PFT-induced death in macrophages was unequivocally blocked by necroptosis inhibition. Based upon these results, we hypothesized that a second cell death signaling pathway was active in PFT-exposed REC.

## Results

### Pore-forming toxins promote the simultaneous activation of caspases and necroptosis during infection

**Figure 1:**
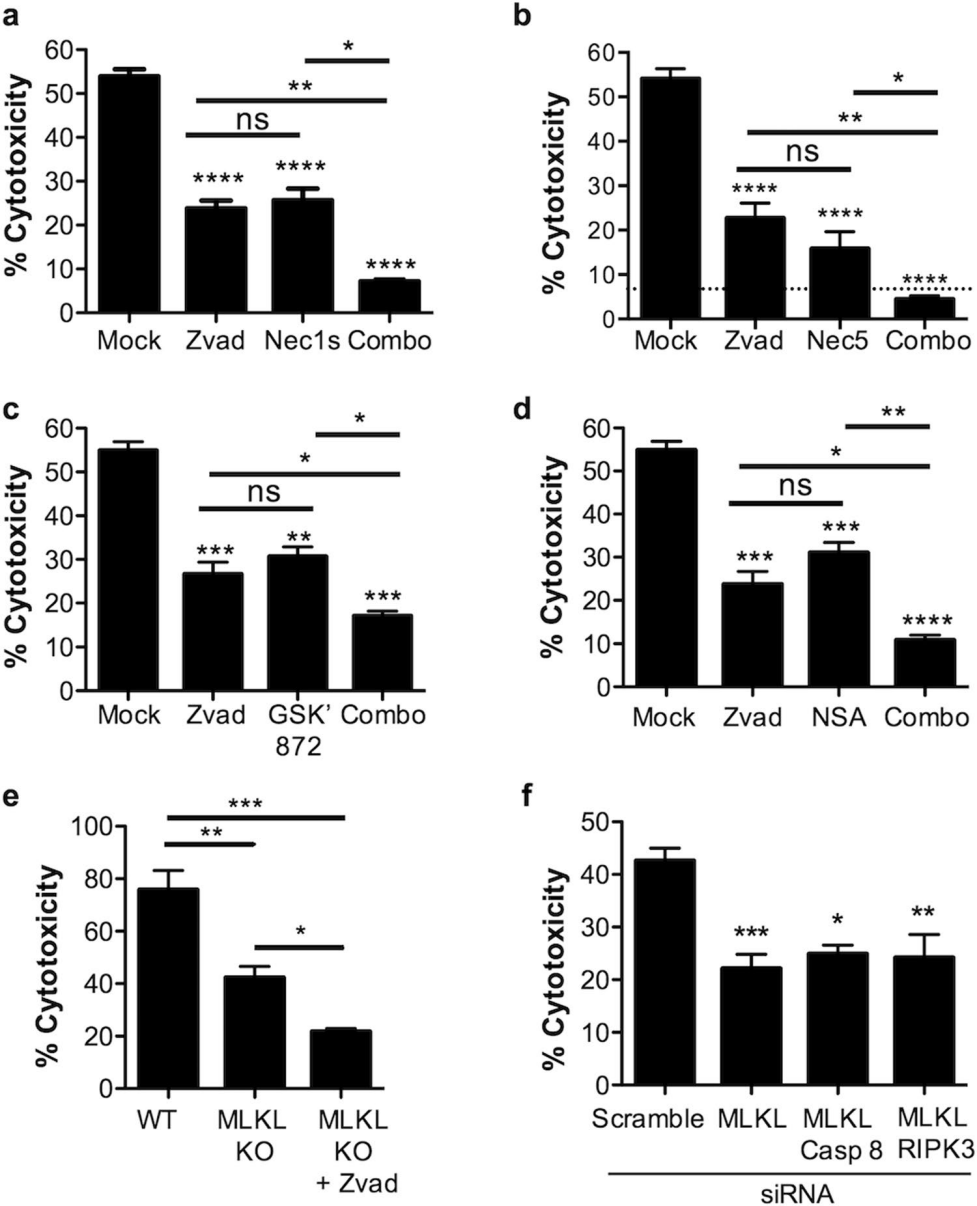
Pore-forming toxins promotes RIPK3-independent activation of caspases and necroptosis to induce cell death. (**a**) LDH release cytotoxicity assay of *Sma* infected A549 cells pre-treated with the pan-caspase inhibitor, Z-VAD-FMK (Zvad, 100 μM), necrostatin-1s (Nec1s, 100 μM), or a combination of Zvad and Nec1s (Combo, 100 μM each). LDH release cytotoxicity assay of *Sma* infected (MOI 10) A549 cells pre-treated with the pan-caspase inhibitor, Z-VAD-FMK (Zvad, 100 μM), (**b**) RIPK1 inhibitor necrostatin-5 (Nec5, 100 μM), (**c**) RIPK3 inhibitor GSK’ 872 (GSK’ 872, 100 μM), (**d**) MLKL inhibitor necrosulfonamide (NSA, 10 μM) or a combination of both (Combo). (**e**) LDH release cytotoxicity assay of wild-type A549 cells or A549 cells deficient in MLKL by CRISPR-Cas9 infected with *Sma* after pretreatment with Z-VAD-FMK or Mock treated. (**f**) LDH release cytotoxicity assay of A549 cells infected with *Sma* following knockdown of MLKL, MLKL/caspase-8 or RIPK3/caspase-8 by siRNA (Blots demonstrating siRNA knock down are shown in Supplementary Figure 3). For multiple group comparisons Kruskal-Wallis test with Dunn’s multiple-comparison post-test was used to test every experimental condition against the mock and between experimental conditions: *P ≤ 0.05, **P ≤ 0.01, ***P ≤ 0.001, ****P ≤ 0.0001. For *in vitro* experiments averaged data from >3 separate experiments are shown.

Pre-treatment of A549 type II pneumocytes with the general caspase inhibitor Z-VAD-FMK (Zvad) and either RIPK1 inhibitor necrostatin-1s (Nec1s) (Fig. 1a), RIPK1 inhibitor necrostatin-5 (Nec5) (Fig. 1b), RIPK3 inhibitor GSK’ 872 (Fig. 1c), or MLKL inhibitor necrosulfonamide (Fig. 1d), all protected in an additive manner versus pretreatment with each drug alone against *Serratia marcescens* (*Sma*) infection. Similar results were observed when A549 cells were pre-treated with Nec-5 and Z-VAD-FMK and challenged with recombinant pneumolysin (rPly) and a similar, very strong, trend with recombinant α-toxin (Supplementary Fig. 1); the latter two being PFTs produced by *Streptococcus pneumoniae* (*Spn*) and *Staphylococcus aureus*, respectively. Conclusively, A549 cells with MKLK deleted using CRISPR-Cas9 experienced additive protection when treated with Zvad (Fig. 1e).

Viral infections have been reported to simultaneously trigger necroptosis and RIPK3/caspase-8 mediated apoptosis within mammalian fibroblasts and lung epithelial cells^23^. Following influenza challenge, RIPK3 has been shown to activate caspase-8^16^. To test for this, we transfected A549 cells with siRNA targeting MLKL and caspase-8 and challenged these with *Sma*. No additive protection was observed in the MLKL and caspase-8 knockdown cells (Fig. 1f, Supplementary Fig. 2). Importantly, no activation of caspases-1, -3 or -8 was observed in *Sma* challenged A549 cells; conversely this was observed when A549 cells were challenged with positive control cyclohexamide or LPS/nigericin (Supplementary Fig. 3). Simultaneous siRNA knockdown of RIPK3 and MLKL also did not have an additive protective effect following *Sma* challenge (Fig. 1f). Together, these results suggest a different form of caspase-associated cell death was occurring in PFT-exposed RECs, one independent of canonical intrinsic or extrinsic apoptosis.

**Figure 2:**
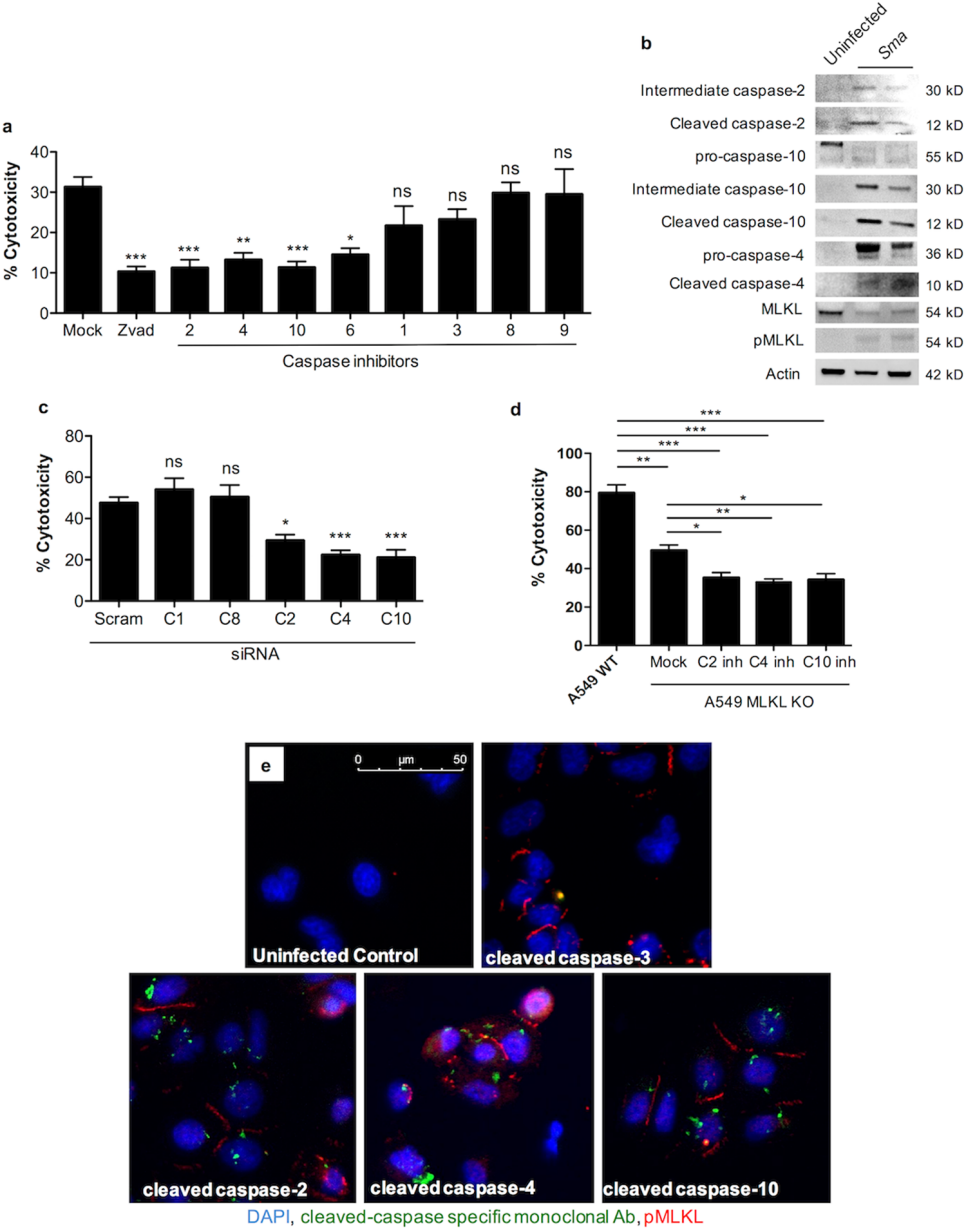
Caspase activity occurs in parallel to necroptosis and can be observed at the single cell level. (**a**) Percent cytotoxicity of *Sma* infected (MOI 0.5) A549 cells pre-treated with Zvad or inhibitors against caspases (C)-1, -2, -3, -4, -6, -8, -9, and -10 (100 μM). Mock infected cells received an equivalent amount of DMSO in media. (**b**) Western blots for the activation of caspase-2, caspase-10, caspase-4, MLKL and pMLKL in A549 cells infected with *Sma*. Blots images were cropped from separate gels, uninfected control (first lane) vs *Sma* infected cells (second and third lane) are shown side-by-side in the same gel. (**c**) LDH release cytotoxicity assay of A549 cells infected with *Sma* following knockdown of caspase-1, caspase-8, caspase-4, caspase-10 and caspase-2 by siRNA. (Blots demonstrating siRNA knock down are shown in Supplementary Figure 6a) (**d**) LDH release cytotoxicity assay of wild-type A549 cells or A549 cells deficient in MLKL by CRISPR-Cas9 infected with *Sma* after pretreatment with inhibitors against caspase-2(C2inh), caspases-4(C4inh) and caspases-10(C10inh) or Mock treated. (**e**) IF Staining for pMLKL, cleaved caspase-2, cleaved caspase-3, cleaved caspase-4 and cleaved caspase-10 in A549 cells challenged with *Sma*. Cell nucleus (DAPI, Blue), caspases (green), pMLKL (Red). For multiple group comparisons Kruskal-Wallis test with Dunn’s multiple-comparison post-test was used: *P ≤ 0.05, **P ≤ 0.01, ***P ≤ 0.001. For *in vitro* experiments averaged data from >3 separate experiments are shown.

At low multiplicity of infection (MOI), chemical inhibition of caspases -2, -4, -6 and -10, but not -1, -3, -8, or -9 was found to confer significant protection against *Sma* (Fig. 2a). At high infectious MOI, protection incurred by inhibition of caspase-6 was lost (Supplementary Fig. 4a). Comparable results were observed when A549 cells were challenged with rPly or α-toxin and when normal human bronchial epithelilal cells (NHBECs) were challenged with *Sma* and rPly (Supplementary Fig. 4b–e). Immunoblots for phosphorylated MLKL and cleaved caspases-2, -4, and -10 showed the active version of these proteins in lysates from A549 cells infected with *Sma* (Fig. 2b). Likewise, A549 cell death was decreased after siRNA knockdown of caspases -2, -4, and -10, but not after siRNA knockdown of caspase-1 and -8 (Fig. 2c, Supplementary Fig. 5a). Again, similar results were also observed after A549 cells were challenge with rPly or α-toxin (Supplementary Fig. 5b,c). Finally, an important role for activation of caspases -2, -4, and -10 following PFT exposure, and one that was not dependent on necroptosis, was suggested after additional protection of MLKL deficient A549 cells against *Sma* when pre-treated with inhitors of these caspases (Fig. 2d).

To verify that caspase activation and necroptosis occurred within the same cell, A549 cells were first examined using propidium iodide (PI), which enters necrotic cells, and then caspase-active specific fluorescent-labeled inhibitors of caspases (FLICA)^25^ after challenge with rPly. We observed that both A549 and NHBECs were PI positive and pan-caspase FLICA positive, providing evidence of ongoing caspase activation and necrosis within the same cell. PI positive cells were also FLICA positive for caspase-2 and -10, but not caspase-1, or -3/7 (Supplementary Fig. 6). Because FLICA may be non-specific, we subsequently examined cells with fluorophore-labeled monoclonal antibodies against cleaved caspase-2, -3, -4, -10, and pMLKL. Consistent with prior results, we observed simultaneous caspase-2, -4 and -10 activation with necroptosis in *Sma* (Fig. 2e). This too was observed in rPly or α-toxin challenged cells (Supplementary Fig. 7).

### Caspase activation during PFT-mediated death changes the morphological characteristics of necroptosis

**Figure 3:**
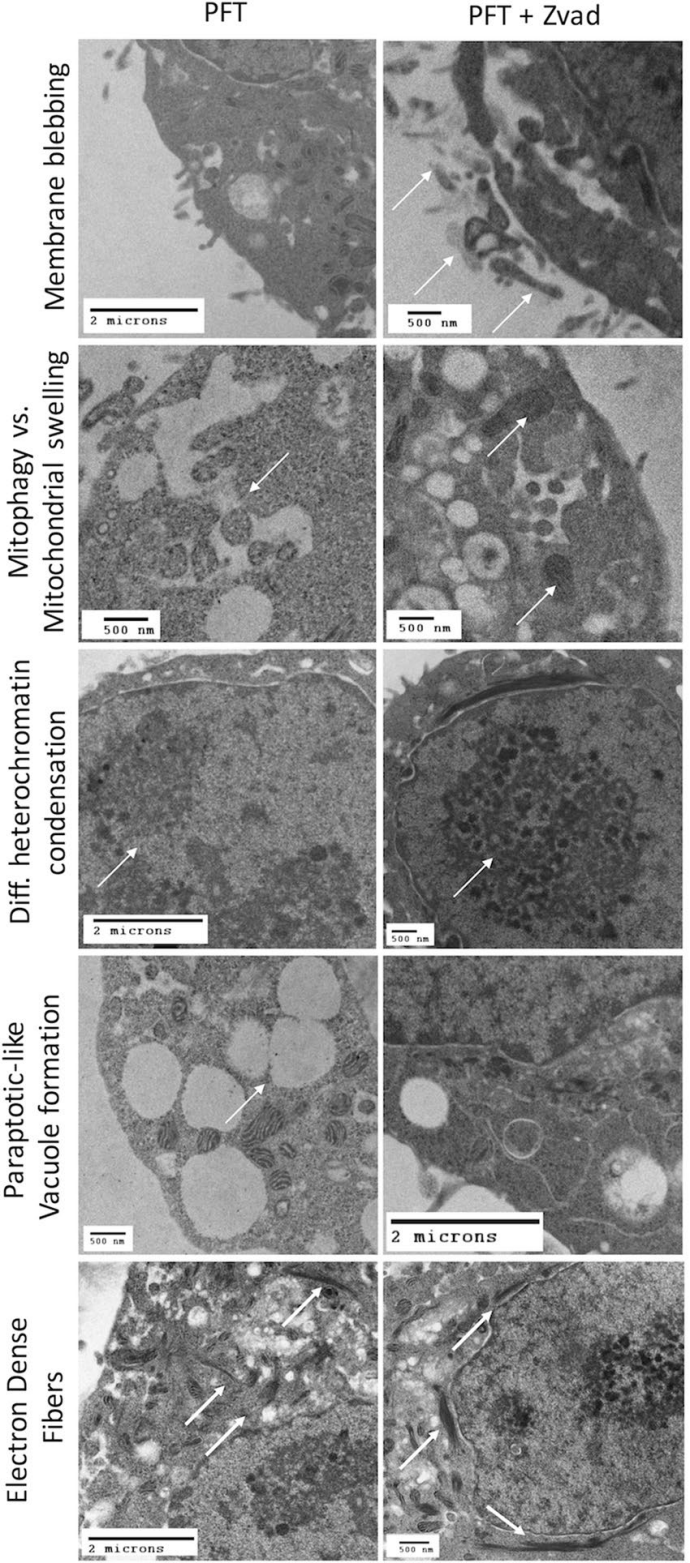
Caspase activity parallel to necroptosis alters PFT-mediated cell death morphology. Representative transmission electron microscopy images of rPly (rPly, 0.32 μg/mL) challenged A549 cells mock treated (for parallel caspase-necroptosis activation) or pre-treated with Z-VAD-FMK (Zvad, for necroptosis, 100 μM). Scale bars with defined dimentions are shown within each picture.

Canonical necroptosis results in cell membrane dissolution, release of cytoplasmic components, and preservation of the nucleus^19^. In contrast, apoptosis involves cell shrinkage, nuclear fragmentation, and the formation of apoptotic bodies^10^. We sought to determine if caspase activity affected the morphological characteristics of cell death in A549 cells treated with pneumolysin in the presence or absence of Zvad (Fig. 3). In cells where caspase activity was not inhibited, transmission electron microscopy images showed less membrane blebbing or “bubbles” than necroptotic cells. In addition, we also observed increased mitophagy, paraptotic-like vacuolation, and heterochromatin condensation in comparison to those undergoing solely necroptosis. In contrast, Zvad treated cells solely undergoing necroptosis showed mitochondrial swelling, singular heterochromatin condensation, and perinuclear electron dense fibers. Interestingly, perinuclear localization of electron dense fibers occurred in greater magnitude in cells treated with Zvad than those with intact caspase activity.

### Caspase activation parallel to necroptosis enhances alarmin release from necroptotic cells and promotes the host inflammatory response

**Figure 4:**
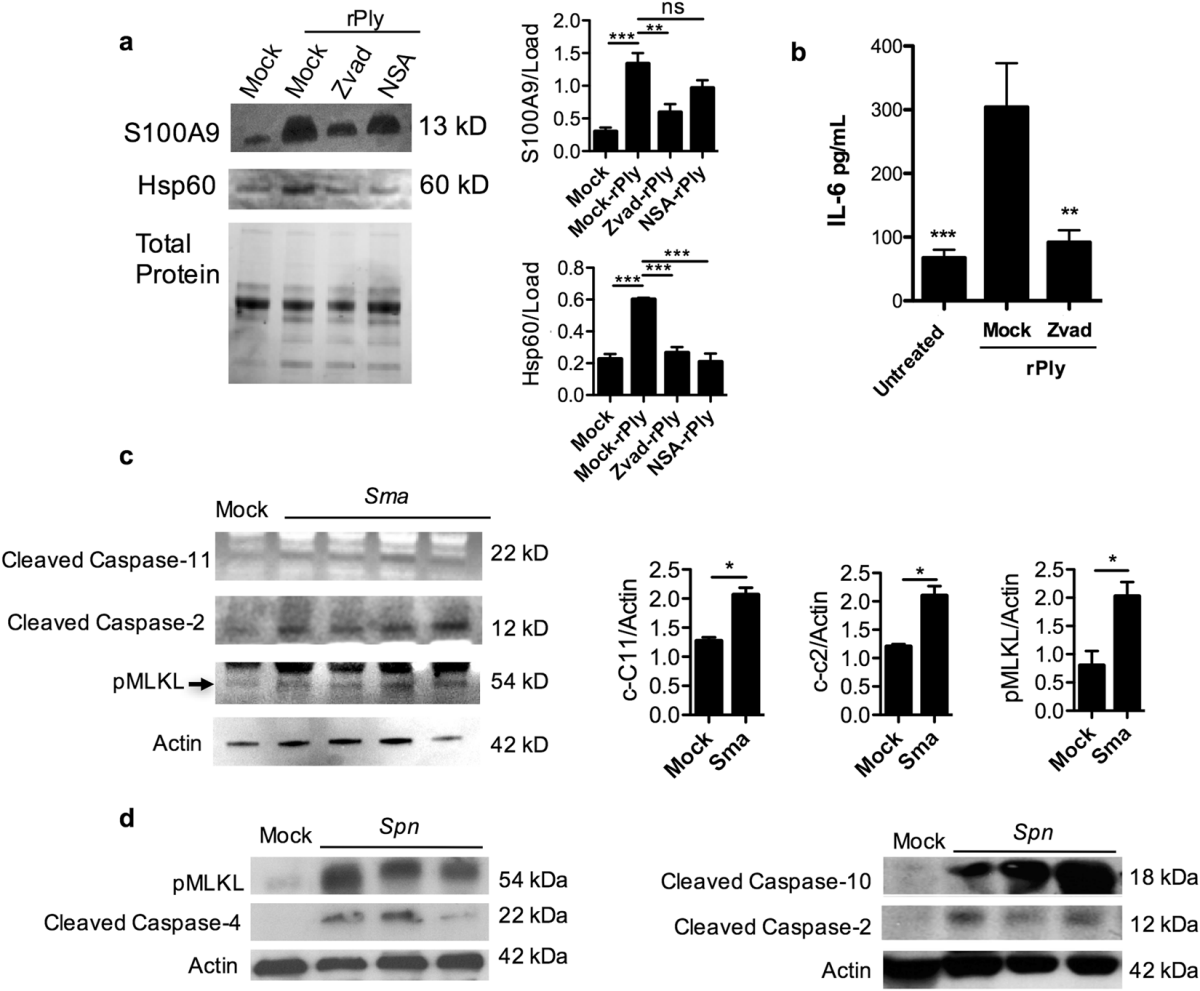
PFT-mediated caspase activity simultaneous to necroptosis promotes alarmin release and inflammation. (**a**) Western blots for S100A9 and HSP60 from A549 cells challenged with rPly after pre-treatment with Z-VAD-FMK (Zvad, 100 μM), or necrosulfonamide (NSA, 10 μM). Blots images were cropped from separate gels, uninfected control (first lane) vs rPly challenged cells (2–4 lanes) are shown side-by-side in the same gel. Densitometry from 3 separate gels is shown next to the immunoblots. (**b**) IL-6 (pg/ml) in supernatants of THP-1 macrophages challenged with the supernatants of A549 cells that were challenged with rPly (subsequently blocked by antibody) pre-treated with Z-VAD-FMK (Zvad, 100 μM), or mock treated. (**c**) Western blot for pMLKL, cleaved caspase-2, and cleaved caspase-4 and actin (loading control) in the lungs of mice (n = 3–4) infected *Sma* via intratracheal route vs uninfected control. Blots images were cropped from separate gels, uninfected control (first lane) vs *Sma* infected mice (2–5 lanes) are shown side-by-side in the same gel. Densitometry from 3 separate gels is shown next to the immunoblots. Extra mock infected controls for densitometry were ran in separate gel. (**d**) Healthy baboons were challenged intrabronchialy with *Spn* using a video assisted bronchoscope and sacrificed 5–7 days after infection for tissue collection. Western blot for pMLKL, cleaved caspase-2, cleaved caspase-4 cleaved caspase-10 and actin (loading control) in the lungs of *Spn* infected baboon’s vs uninfected control. Blots images were cropped from separate gels, uninfected control (first lane) vs *Spn* infected baboon’s (2–4 lanes) are shown side-by-side in the same gel. For multiple group comparisons Kruskal-Wallis test with Dunn’s multiple-comparison post-test was used: *P ≤ 0.05, **P ≤ 0.01, ***P ≤ 0.001. For *in vitro* experiments averaged data from >3 separate experiments are shown.

Due to the observed differences described above, we also hypothesized that caspase activation parallel to necroptosis modulates the alarmins that are released from dying cells. To test this, we pretreated A549 cells with Zvad or the NSA, and measured levels of known alarmins released into the cell supernatants following challenge with rPly. We found that caspase activity promoted the release of S100A9 and Hsp60 into the cell supernatants (Fig. 4a). To assess the effect of caspase-mediated alarmin release on bystander immune cells, we challenged mock or Zvad treated A549 cells with rPly and collected their supernatants. Subsequently, the activity of residual rPly was neutralized with a monoclonal antibody^26^ and the supernatants from these cells were used to challenge human macrophages (THP-1 cells). We found that IL-6 production by human THP-1 macrophages was reduced when exposed to supernatants from cells that had caspase activity inhibited during PFT-induced necroptosis (Fig. 4b).

**Figure 5:**
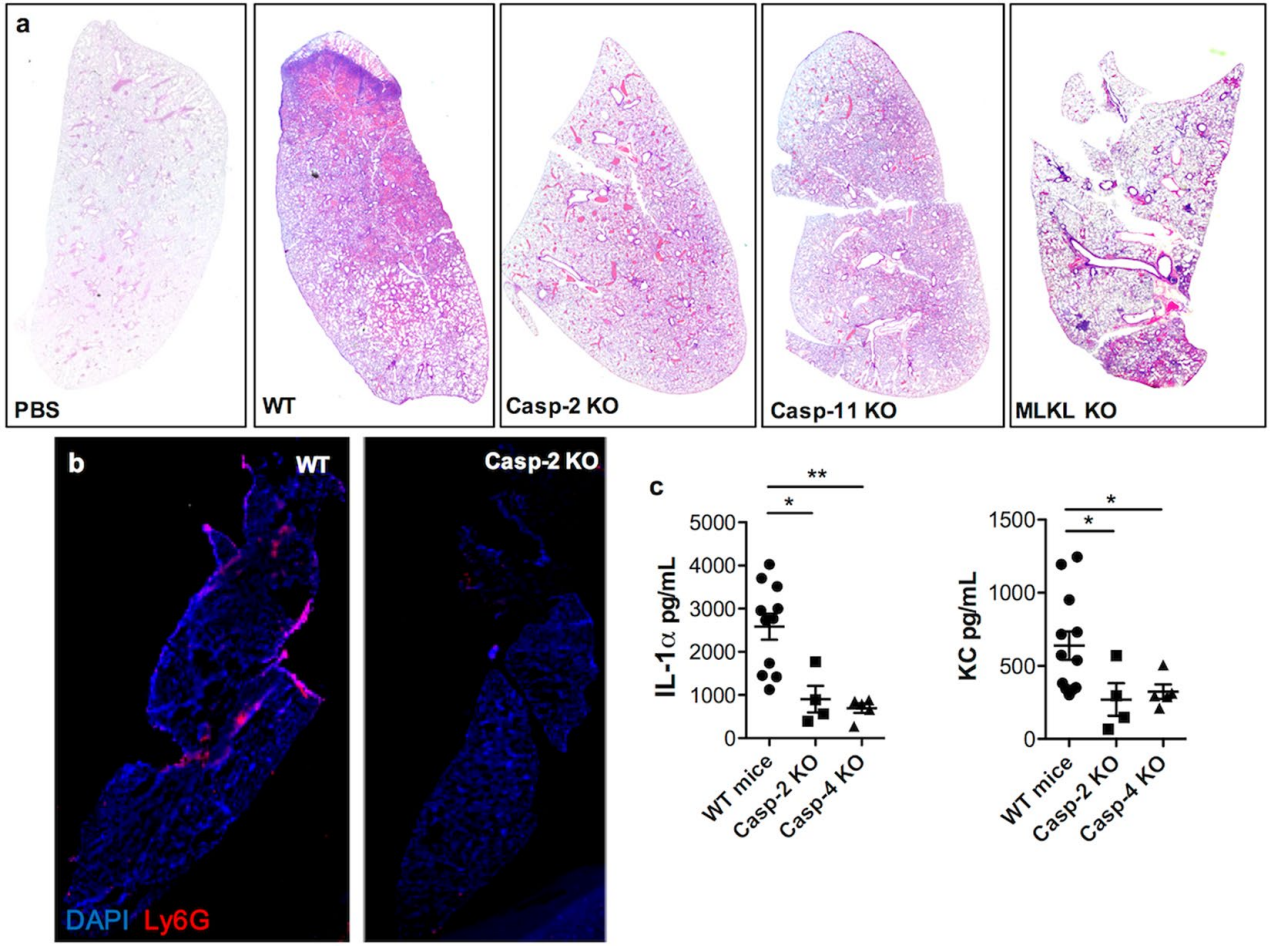
Caspase-2 and -11 KO reduces PFT-mediated inflammation during bacterial pneumonia. (**a**) Hematoxylin and Eosin stain of mice genetically deficient in MLKL, caspase-2 and caspase-4 experiencing *Sma* pneumonia or mock challenged with PBS. (**b**) Representative IF staining of lungs of caspase-2 KO mice infected with *Sma*, cell nucleus (DAPI, Blue), Ly6G (red). (**c**) Levels of IL-1α and KC in lung homogenates from wild-type, caspase-2 KO and caspase-4 KO mice infected with *Sma*. For multiple group comparisons Kruskal-Wallis test with Dunn’s multiple-comparison post-test was used: *P ≤ 0.05, **P ≤ 0.01, ***P ≤ 0.001. For *in vitro* experiments averaged data from >3 separate experiments are shown.

Validating this *in vivo*, we observed increased levels of cleaved caspase-2, cleaved caspase-11, and pMLKL in lung samples from mice challenged intratracheally with *Sma* (Fig. 4c, Supplementary Fig. 8a). Whereas in lung samples from non-human primates with experimental *Spn* pneumonia we also observe increased levels of cleaved caspase-2, -4 and -10 along with pMLKL (Fig. 4d, Supplementary Fig. 8b). Critically, mice deficient in caspase-2 or caspase-11 that had been challenged with *Sma* showed a stark reduction in lung consolidation when compared to wildtype, similar to mice deficient in MLKL (Fig. 5a). Immunofluorescent staining against Ly6G, a canonical marker of mature neutrophils, showed a substantial decrease in granulocyte infiltration into the lungs (Fig. 5b, Supplementary Fig. 8c) that coincided with reduced Interleukin-1α and KC levels in lung homogenates (Fig. 5c). Thus, caspase activation worsened severity of pneumonia.

## Discussion

REC play many critical immunological roles during bacterial infection; they serve as a physical barrier, secrete mucus and anti-microbial molecules, and produce chemokines that recruit immune cells to the site of infection^27,28^. In the recent past we have shown that PFTs cause necroptosis of REC and this is, in large part, responsible for the pulmonary injury that occurs during pneumonia^24^. Herein, we demonstrate that upon challenge with PFTs, RECs undergo necroptosis with parallel caspase activity and this potentiates the inflammatory response by enhancing the release of alarmins from dying cells. Our results raise questions and open new lines of investigation that can improve our understanding of basic mechanisms of infectious disease and the host response. For example, what other cell types experience parallel caspase activation to necroptosis following exposure to a PFT and why is this not the case for macrophages? Is there a PFT-mediated necroptosis-caspase signaling intersection where MLKL potentiates caspase activity, albeit these pathways seem independent? These are critical question that now require detailed exploration.

One overriding question is that it is unknown how these caspases are being activated. Caspase-2 can function as an initiator and/or effector caspase, a capability that matches its ancient and conserved function^17^. Caspase-2 might, in some fashion, recognize the binding of PFTs to the plasma membrane or the associated consequences^14^. In this manner, caspase-2 may aggregate, oligomerize, and autoprocess itself upon PFT challenge of cells^29^. It is important to note that caspase-2 has been shown not to process canonical apoptosis effectors such as caspases -3, -6 or -7^30^. Instead it leads to the induction of apoptosis by increasing the release of death mediators from the mitochondria^30^. In addition, caspase-2 has been shown to be involved in cell death mediated by cellular stress such as DNA damage, ER stress, or metabolic catastrophe^31^. A review of the literature also suggests that targeting of the mitochondria by MLKL, or even by the PFT itself, may activate mitochondrial localized caspase-2^32^. Other substrates released from damaged mitochondria include BID, ICAD, and PKCδ which promote mitochondrial outer membrane permeabilization activation and DNA fragmentation^29^, and all of which are known caspase-2 substrates or initiating signals^29^. For these reasons, we speculate that RIPK activation might further promote caspase activation upon MLKL-driven mitochondrial damage^33^ although this association would not be required for necroptosis.

Our data using human cells and baboon samples show that caspase-10 is active upon PFT challenge. Caspase-10 is a homolog of caspase-8, and is highly conserved throughout multiple species, however absent in mice. Caspase-10 has been shown to have a key role in death receptor-mediated apoptosis^34^. Importantly, it has been shown that caspase-10 reduces death-inducing signaling complex (DISC) association and activation of caspase-8, by this it negatively regulates caspase-8 mediated death^35^. Notably, for necroptosis to occur, caspase-8 has to be inhibited, suggesting that upon PFT challenge the activation of caspase-10 might promote necroptosis and at the same time serve as an initiator for other death mediators. In addition, caspase-10 activation through a membrane receptor could also explain the activation of caspase-2. However, the latter has yet to be elucidated.

Caspase-4 is an inflammatory caspase, recently linked to the non-canonical activation of the inflammasome and the release of IL-1β. This is known to occur after cells are exposed to intracellular lipopolysaccharide^36^. PFTs might be a way to introduce LPS into the cytosol during Gram-negative infections. However, we observed caspase-4 activation in cells challenged with purified PFTs (tested to be endotoxin free). One possible reason caspase-4 is activated in an LPS-independent manner may be endoplasmic reticulum stress caused by PFT-induced ion dysregulation, cellular ROS generation, and uncontrolled metabolic changes^5,24^. Endoplasmic reticulum stress has been implicated as an activator of caspase-4 in other model systems^37,38^. Regardless, activation of caspase-4, and presumably the inflammasome, would allow for respiratory epithelial cells to produce and release pro-inflammatory mediators during PFT-mediated necroptosis. Whether this occurs is currently being investigated.

How caspase activity acts on organelle disassembly to impact alarmin release is not clear. Yet, caspase-inhibited REC challenged with rPly had obvious differences in cellular traits during death versus controls, and released less alarmins. That caspase-activation further promoted necroptosis-driven inflammation in the airway was also unambiguous. This was evidenced *in vitro*, where naïve macrophages treated with cell supernatants from caspase inhibited REC responded less vigorously than controls, as well as *in vivo*, were we observed that caspase-2 and caspase-11 KO mice had reduced tissue damage and levels of measurable inflammatory chemokines and cytokines. The recognition that caspase activation contributes to pulmonary inflammation marks the involved caspsases as potential targets for adjunct therapeutic intervention along with antimicrobials. In this instance not to clear the pathogen, but to reduce lung injury due to the excessive host response.

Herein, we have discovered that caspase activity, other than caspase-8, can occur parallel to the necroptosis machinery. Caspase activation may occur as an evolutionary conserved response to PFTs during pulmonary infections^14,29,39,40^. In the airway, caspase activity during necroptosis was observed to exacerbate pulmonary inflammation by modulating the physical properties of cell death, which in turn impacted alarmin release. The almost universal presence of PFTs in bacterial pathogens makes this discovery highly significant and identifies caspases as potential targets for novel intervention strategies. Numerous unanswered question remain as to how caspase activation is initiated and whether it is intertwined with necroptosis signaling. Which other cells and diseases might include a version of this form of cell death is also unknown and requires further investigation.

## Methods

### Ethics Statement

Animal experiments were performed using protocols approved by the Institutional Animal Care and Use Committee at the University of Alabama at Birmingham (Protocol # 20270). Animal care and protocols followed the NIH Guide for the Care and Use of Laboratory Animals.

### Infections of Mice and Baboons

Female 6–8-week-old wildtype C57BL/6 mice from Jackson Laboratories (Sacramento, California) were used. MLKL knockout mice in the C57BL/6 background were obtained from Dr. Warren Alexander^41^. Caspase-2 KO (007899) and Caspase-11 KO (024698) mice were obtained from The Jackson Laboratory (Sacramento, California). Oropharyngeal aspiration was performed on each mouse as previously described with an inoculum of 100 μl of a ~1.0 × 10^6^ CFU dose^42^. Briefly, after being anesthetized (2% vaporized isoflurane) each mouse was hung upright by its incisors, the tongue gently pulled outward with blunt forceps, and the respective inoculum pipetted into the pharynx accompanied by coverage of the nares to achieved forced inhalation. All mice experiments were performed with protocols approved by the University of Alabama at Birmingham Institutional Animal Care and Use Committee and in agreement with the NIH Guide for the Care and Use of Laboratory Animals. Healthy baboons, with a median age 11 (Interquartile radio [IQR], 10–19) years old were intrabronchialy challenged with *Spn* (1 × 10^9^ CFU) using a bronchoscope^43,44^. Between 5–7 days after infection during severe pneumonia animals were sacrificed and lung tissue was collected. All baboon experiments were performed using protocols approved by the Southwest National Primate Research Center Institutional Animal Care and Use Committee and in agreement with the NIH Guide for the Care and Use of Laboratory Animals.

### Microscopy and Image Capture

Images were captured using a Zeiss AxioXam MRm Rev3 and/or MRc cameras attached to a Zeiss AxioImager Z1 epifluorescent microscope (Carl Zeiss, Thornwood, NY) or a Leica LMD6 with DFC3000G-1.3-megapixel monochrome camera (Leica Biosystems, Buffalo Grove, IL).

### Inhibitors and other chemicals

RIPK1 inhibitor Necrostatin-5 was obtained from Sigma Aldrich (St. Louis, MO). Necrostatin-1s and GSK’872 were obtained from BioVision (Milpitas, California). Necrosulfonamide was obtained from Tocris Bioscience (QL, United Kingdom). To inhibit caspases, we used Z-VAD-fmk, general caspase inhibitor, Z-WEHD-fmk, caspase-1 inhibitor, Z-VDVAD-fmk, caspase-2 inhibitor, Z-DEVD-fmk, caspase-3 inhibitor, Z-YVAD-fmk, caspase-4 inhibitor, Z-VEID-fmk, caspase-6 inhibitor, Z-IETD-fmk, caspase-8 inhibitor, Z-LEHD-fmk, caspase-9 inhibitor, Z-AEVD-fmk, caspase-10 inhibitor, obtained from R & D Systems (Minneapolis, MN).

### Transmission electron microscopy (TEM)

Published protocols were used for tissue processing^45^. TEM images were obtained at the University of Alabama at Birmingham electron microscopy core, using FEI-Tecnai T12 Spirit 20–120kv, equipped with an AMT digital camera.

### Silencing RNA and CRISPR-Cas9

Commercially available siRNA targeting RIPK1, RIPK3, MLKL, caspase-1, caspase-2, caspase-4, caspase-8 and caspase-10 (Santa Cruz, Dallas, TX) were used to transfect A549 alveolar epithelial cells following the manufacturer instructions. Commercially available CRISPR-Cas9 plasmids to knock out MLKL (Santa Cruz, Dallas, TX) were used following the manufacturer instructions.

### Statistical analysis

Prism 7 (Graph Pad Software, La Jolla CA) was used for graph development and statistical analysis. Mann-Whitney U tests were applied for two-group comparisons, and nonparametric Kruskal-Wallis test and Dunn’s *post hoc* analysis were used for multiple-group comparisons.

### Data availability

The data generated in the current study are available from the corresponding author on reasonable request.

## Electronic supplementary material

Supplementary Information
