# Supplementary material for: Bacterial Pore-Forming Toxins Promote the Activation of Caspases in Parallel to Necroptosis to Enhance Alarmin Release and Inflammation During Pneumonia
Source: Sci Rep. 2018 Apr 11;8:5846. doi: 10.1038/s41598-018-24210-8 (PMC5895757; doi:10.1038/s41598-018-24210-8)
Supplement: Supplementary file 1 — Supplementary Information [file 41598_2018_24210_MOESM1_ESM.pdf]

Title: Bacterial Pore-Forming Toxins Promote the Activation of Caspases in Parallel to Necroptosis to Enhance Alarmin Release and Inflammation During Pneumonia.

Authors: Norberto Gonzalez-Juarbe[1]*, Kelley M. Bradley[1], Ashleigh N. Riegler[1], Luis F. Reyes[2], Terry Brissac[1], Sang-Sang Park[1], Marcos I. Restrepo[2], and Carlos J. Orihuela[1]*

Authors Affiliations: [1]Department of Microbiology, The University of Alabama at Birmingham, 845 19th Street South, Birmingham, Alabama 35294-2170, USA. [2]Division of Pulmonology, South Texas Veterans Health Care System, San Antonio, TX 78229.

***Corresponding Authors:** Drs. Norberto González-Juarbe and Carlos J. Orihuela, Department of Microbiology, University of Alabama at Birmingham, BBRB 667, 1720 2nd Avenue South, Birmingham, AL, 35294-2170, emails: norberto@uab.edu, corihuel@uab.edu

SUPPLEMENTAL INFORMATION

Supplemental Figures:

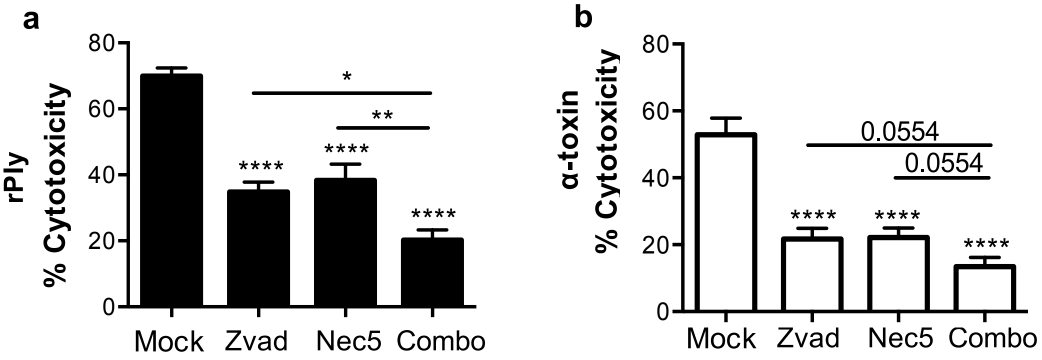

Supplemental Figure 1: Caspase activation occurs during PFT-induced necroptosis in A549 Cells. LDH release cytotoxicity assay of *Sma* infected (MOI 10) A549 cells pre-treated with the pan-caspase inhibitor, Z-VAD-FMK (Zvad, 100µM), necrostatin-5 (Nec5, 100µM), or a combination of both (Combo), challenged with purified (**a**) *S. pneumoniae* pneumolysin (rPly, 0.32µg/mL) and (**b**) *S. aureus* α-Tox (3.9µg/mL). For multiple group comparisons Kruskal-Wallis test with Dunn's multiple-comparison post-test was used: *, P ≤ 0.05, **, P ≤ 0.01, ***, P ≤ 0.001, ****, P ≤ 0.0001 to test every experimental condition against the mock and between experimental conditions. For *in vitro* experiments averaged data from >3 separate experiments are shown.

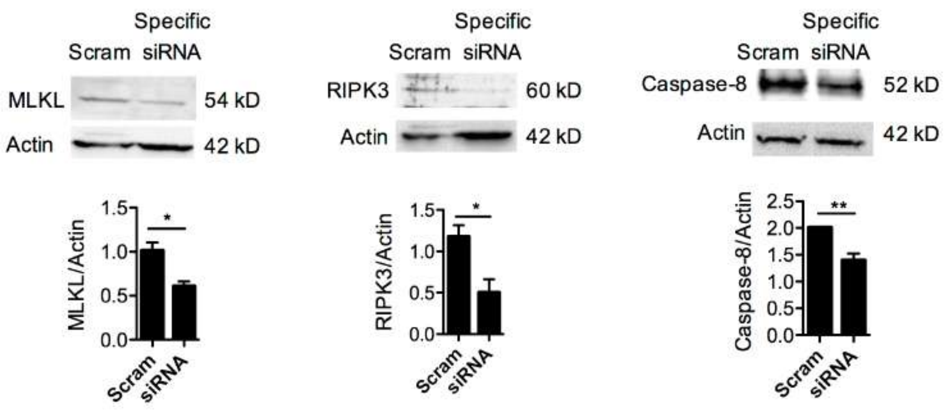

Supplemental Figure 2: siRNA knockdown of MLKL, RIPK3 and caspase-8. A549 alveolar epithelial cells were transfected with siRNA targeting MLKL, RIPK3, and caspase-8 or with a scrambled control. Western blots for MLKL, RIPK3, and caspase-8 after specific siRNA transfection of A549 cells for data shown on figure 1f, blots images were cropped from separate gels, scramble siRNA control (first lane) vs selective siRNA (second lane) are shown side-by-side. Densitometry from 3 separate gels is shown next to the immunoblots. Unpaired t test was used to analyze groups due to the small sample size.

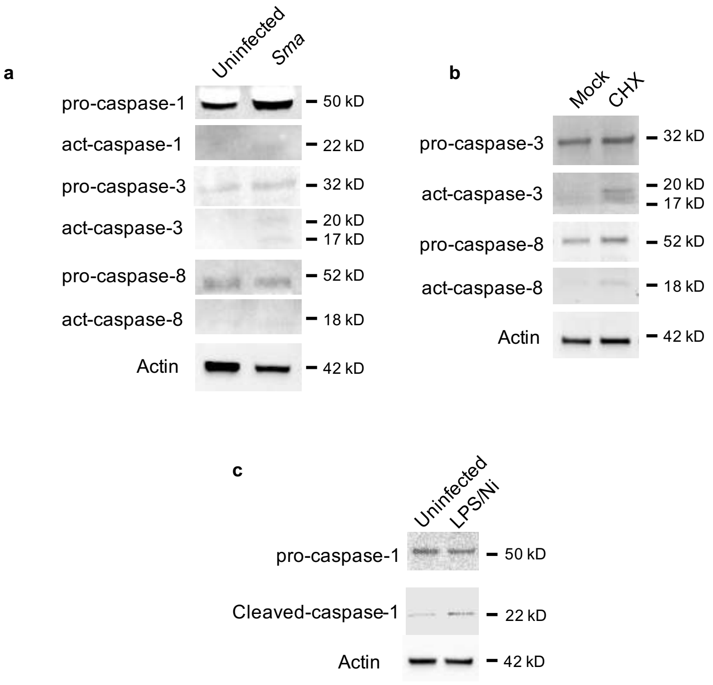

Supplemental Figure 3: Caspases -1, -3 and -8 are not required during caspase-associated necroptosis. (**a**) Western blots for the activation of caspase-1, caspase-3, and caspase-8 in A549 cells infected with *Sma*. (**b**) Western blots for the activation of caspase-3, and caspase-8 after treatment ofA549 cells with cyclohexamide (CHX) (apoptosis). (**c**) Western blots for the activation of caspase-3, and caspase-8 after treatment of A549 cells with nigericin and LPS (pyroptosis). Blots images were cropped from separate gels, Uninfected control (first lane) vs experimental challenge (second lane) are shown side-by-side.

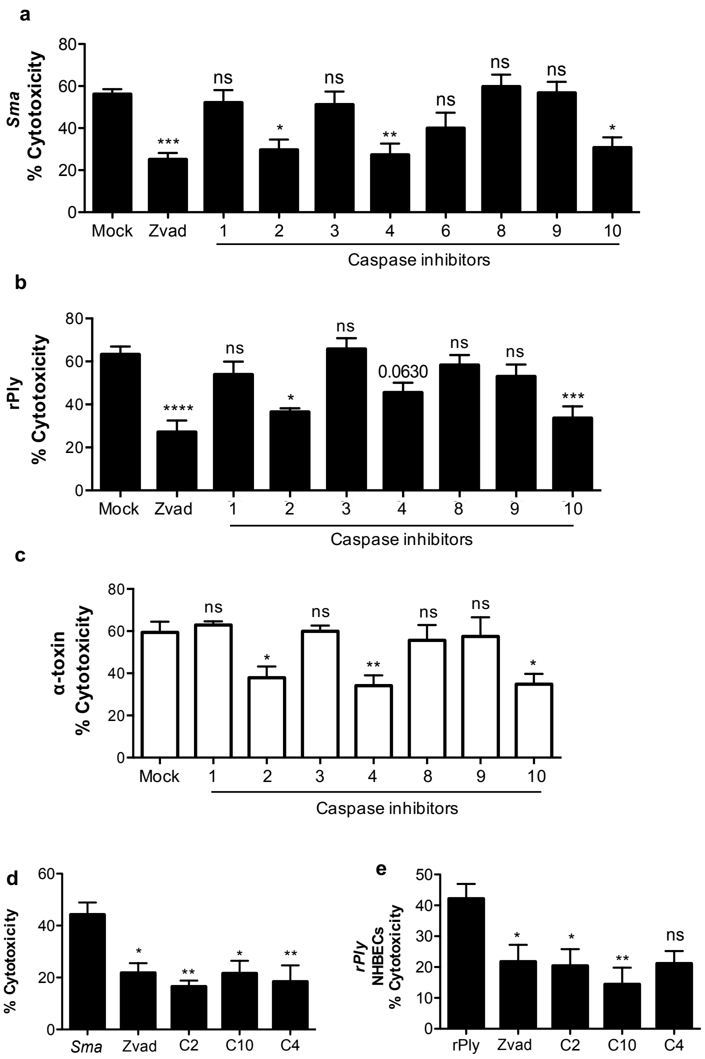

Supplemental Figure 4: Caspases -2, -4 and -10 activity are active during alveolar epithelial necroptosis. (**a**) LDH release cytotoxicity of assay of *Sma* infected (MOI 10) A549 cells pre-treated with Zvad or specific inhibitors against caspases (C)-1, -2, -3, -4, -6, -8, -9, and -10 (100 µM). Mock infected cells received an equivalent amount of DMSO in media. LDH release cytotoxicity assay of A549 cells pretreated with specific inhibitors against caspase (C)1, 2, 3, 4, 8, 9, 10 and challenged with purified (**b**) rPly (0.32µg/mL) or (**c**) *S. aureus* α-toxin (3.9µg/mL). (**d**) LDH release cytotoxicity assay of Normal Human Bronchial Epithelial Cells (NHBEC) pre-treated with the general caspase inhibitor, Z-VAD-fmk (Zvad, 100µM), or inhibitors of caspase-2 (C2), caspase-4 (C4) and caspase-10 (C10) (all at: 100µM) infected with *Sma* (MOI 10). (**e**) LDH release cytotoxicity assay of NHBEC pre-treated with the general caspase inhibitor, Z-VAD-fmk (Zvad, 100µM), or inhibitors of caspase-2 (C2), caspase-4 (C4) and caspase-10 (C10) (all at: 100µM) challenged with pneumolysin (rPly, 0.32µg/mL). For multiple group comparisons Kruskal-Wallis test with Dunn's multiple-comparison post-test was used: *, P ≤ 0.05, **, P ≤ 0.01, ***, P ≤ 0.001, ****, P ≤ 0.0001. For *in vitro* experiments averaged data from >3 separate experiments are shown.

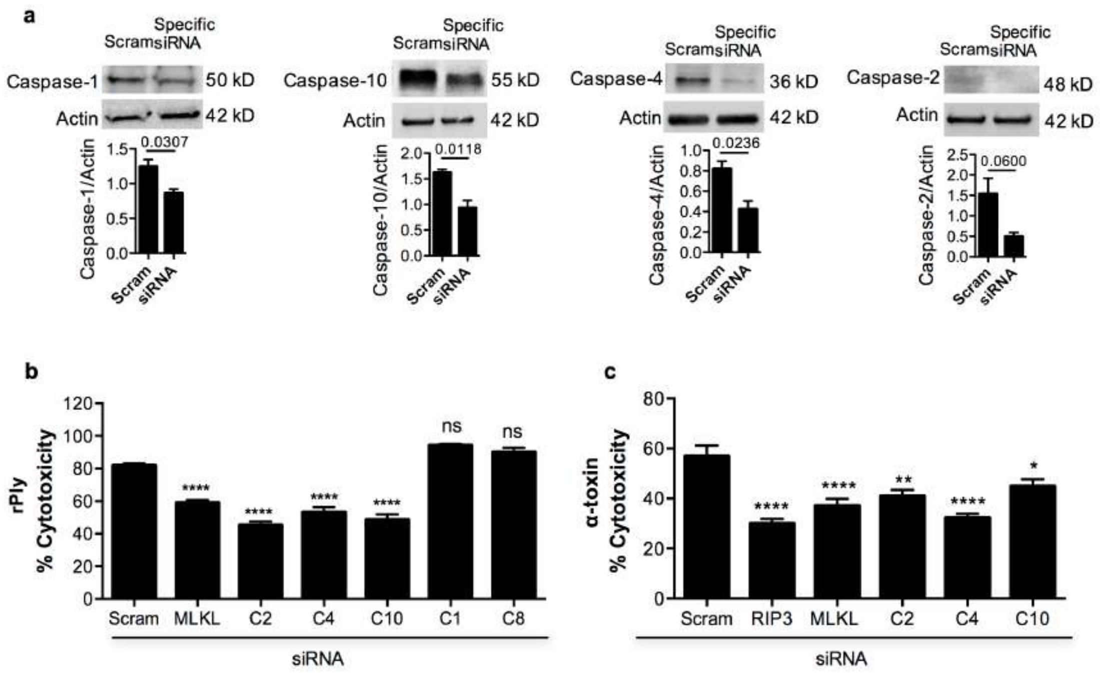

Supplemental Figure 5: siRNA knockdown of caspases -2, -4 and -10 is protective against PFT mediated cell death. (**a**) A549 alveolar epithelial cells were transfected with siRNA targeting caspase-1, caspase-10, caspase-4 and caspase-2 or with a scrambled control. Western blots for MLKL, RIPK3, and caspase-8 after specific siRNA transfection of A549 cells for data shown on figure 1f, blots images were cropped from separate gels, scramble siRNA control (first lane) vs selective siRNA (second lane) are shown side-by-side. Densitometry from 3 separate gels is shown next to the immunoblots. Unpaired t test was used to analyze groups due to the small sample size. (**b-c**) LDH release cytotoxicity assay of A549 cells infected challenged with (**b**) rPly (0.32μg/mL) or (**c**) *S. aureus* α-toxin (3.9μg/mL) following knockdown of MLKL, RIPK3 (RIP3), caspase-1, caspase-2, caspase-4, caspase-8 and caspase-10 by siRNA. For multiple group comparisons Kruskal-Wallis test with Dunn's multiple-comparison post-test was used: *, $P \leq 0.05$, **, $P \leq 0.01$, ***, $P \leq 0.001$, ****, $P \leq 0.0001$. For *in vitro* experiments averaged data from >3 separate experiments are shown.

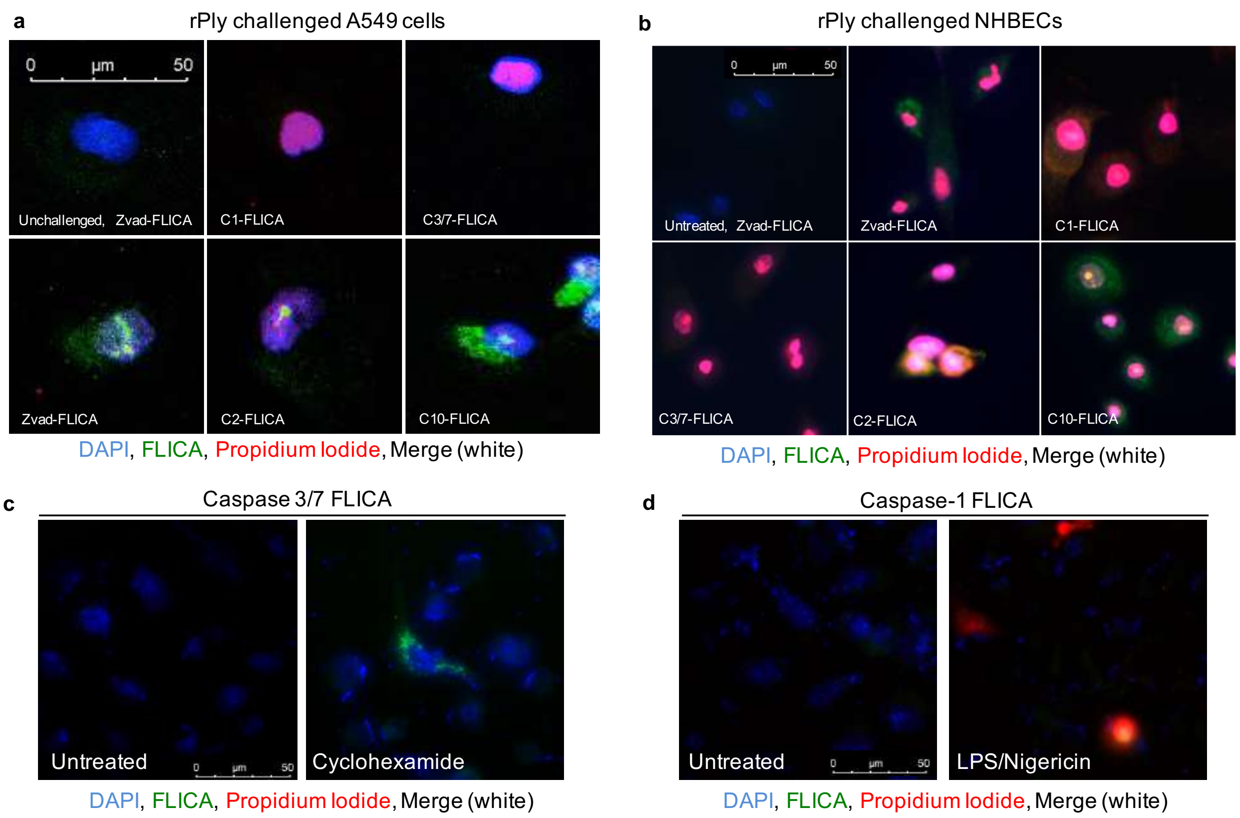

Supplemental Figure 6: Fluorescent-labeled Inhibitor of Caspases staining shows caspase activation in necrotic cells. Caspase activity measured by fluorescent-labeled inhibitor of caspases (FLICA-green) for all caspases (Zvad-FLICA), caspase-1 (C1-FLICA), caspase-2 (C2-FLICA), caspases-3/7 (C3/7- FLICA), caspase-10 (C10-FLICA) and an untreated control stained with Zvad-FLICA. (**a**) A549 cells challenged with rPly (0.32µg/mL). (**b**) NHBEC infected with rPly (0.32µg/mL). (**c**) A549 cells challenged with cyclohexamide (CHX) (apoptosis). (**d**) A549 cells challenged with nigericin and LPS (pyroptosis). Propidium Iodide (red) stained cells undergoing necrosis and DAPI stained nucleic DNA (blue).

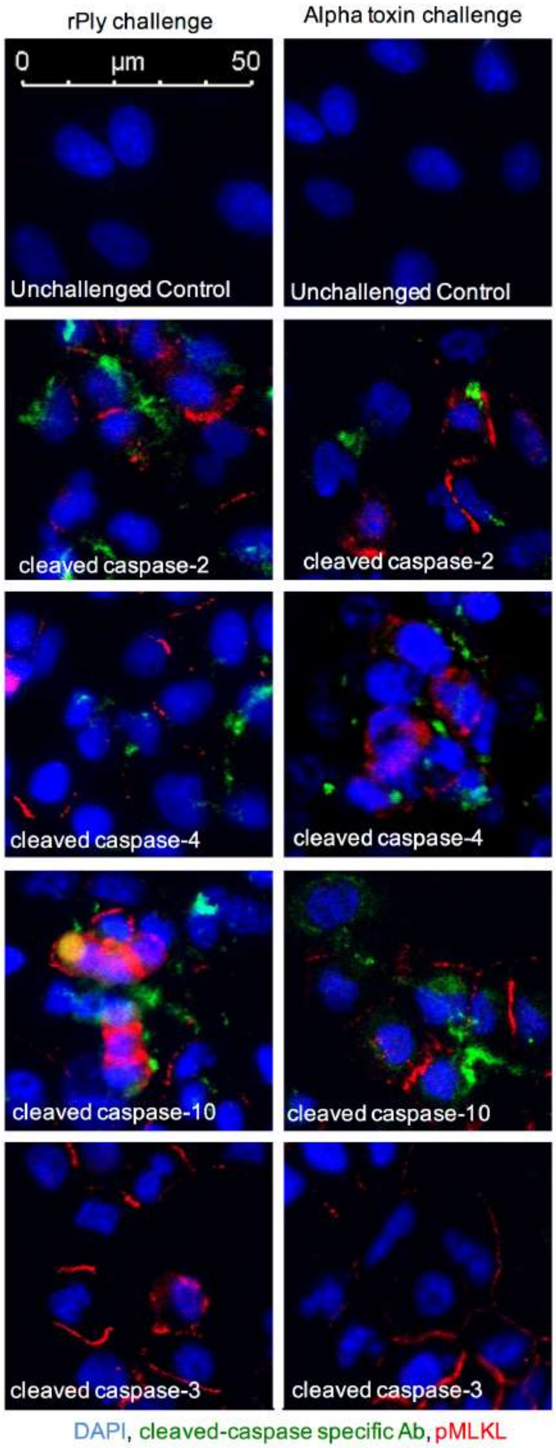

Supplemental Figure 7: Caspase activation occurs parallel to MLKL activation. IF Staining for pMLKL, cleaved caspase-2, cleaved caspase-3, cleaved caspase-4 and cleaved caspase-10 in A549 cells challenged with rPly (0.32µg/mL) or *S. aureus* α-toxin (3.9µg/mL). Cell nucleus (DAPI, Blue), caspases (green), pMLKL (Red).

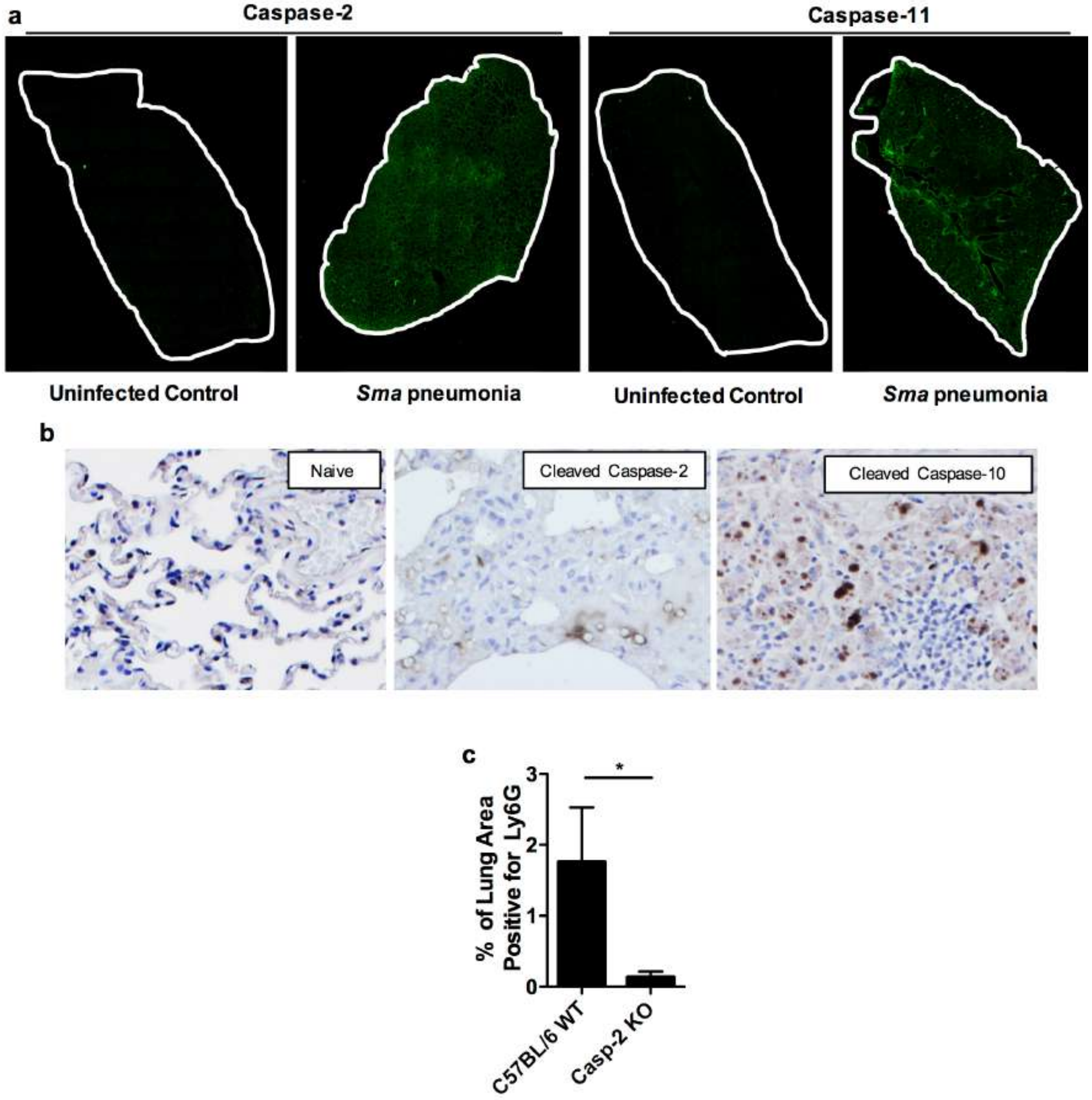

Supplemental Figure 8: Caspase-2 and -10 activity occurs mice and non-human primates and promotes inflammation. (**a**) Representative immunohistochemistry staining for active caspases-2 and -10 in non-human primates infected with s. pneumoniae. (**b**) Representative immunofluorescent staining for active caspase-2 and -10 in lungs of mice infected with *Sma*. (**c**) Quantification of Ly6G signal from whole lungs infected with *Sma* (n=4 mice), representative image Figure 4f. Mann-Whitney U tests were applied for two-group comparisons: *, P ≤ 0.05.

Supplemental Experimental Procedures:

Bacterial Strains and Growth Conditions. A clinical isolate of *S. marcescens* (*Sma*) was obtained from Dr. James Jorgensen at the University of Texas Health Science Center at San Antonio, San Antonio Texas. *Sma* was grown on Luria-Bertani (LB) agar plates and incubated overnight at 30˚C. A single colony was transferred to LB broth and incubated at the same temperature O/N with rolling, then back diluted 1:50 for 3 additional hours (1). *S. pneumoniae* TIGR4 wild type (*Spn*) was grown as previously described (2). Recombinant pneumolysin (rPly) was purified from transformed *E. coli* also as previously described (2). Purified *Staphylococcus aureus* alpha-toxin was obtained from Sigma-Aldrich. rPly concentration used was previously published to cause cardiomyocyte and respiratory epithelial cell cytotoxicity (2, 3) and both rPly and alpha-toxin were further tested using in a hemolysis assay and found to cause 75% lysis in red blood cells at 0.32 ug/ml and 3.9 ug/ml, respectively. The inoculums were prepared by diluting the bacteria into sterile phosphate-buffered saline (PBS) to the desired final concentration (100µL volume inoculum). The amount of colony forming units (CFU) inoculated was confirmed at the time of infection by serial dilution of the inoculum, plating on agar plates, and extrapolation from colony counts.

Cell Death Assays. Cell death was evaluated by detection of the cytoplasmic enzyme lactate dehydrogenase (LDH) in the culture supernatants as previously described (4). Briefly, cells were spun down at 300xg for 5 minutes and 50µL supernatant was removed from each well and passed to a new 96-well plate, and immediately assayed for the presence of LDH using the Cytotox 96 Assay kit (Promega, Madison, WI) or Pierce LDH cytotoxicity kit (Thermo Fisher Scientific, Suwanee, GA) according to the manufacturer's recommendations. Note that positive controls cells were treated with lysis buffer to liberate all cytoplasmic content approximately 30 minutes prior to supernatant harvest. 50µL of reconstituted substrate solution was added to the supernatants, and then incubated for 30 minutes in the dark. The addition of 5µL of stop solution provided by manufacturer terminated the reaction. Absorbance was measured at 490nm in a BioTek Synergy H4 plate reader (BioTek, Winooski, VT) or iMark Absorbance Microplate Reader (Bio-Rad Laboratories, Hercules, CA). Media controls incubated in the presence of the drugs and chemicals used in this study showed that they did not affect LDH detection.

Histology. After euthanasia by pressurized CO_2 asphyxiation and pneumothorax, lungs were inflated with 0.5mL of optimal cutting medium (Tissue-Tek OCT, Sakura, Torrance CA), surgically removed, transferred to molds and snap-frozen covered in OCT. Tissue sections were stained with hematoxylin and eosin or by immunofluorescent stains as previously described (1, 3).

***In Vitro* Infections.** For *in vitro* studies, bacterial cells were grown as described above. After back dilution, bacteria were added to adherent mammalian cells at a multiplicity of infection (MOI) 10 (*S. marcescens*), 100 (*S. pneumoniae*) in their respective media without phenol red + 2% FBS for up to 4 hours. For inhibition or protection experiments, cells were first pretreated with various concentrations of selected chemicals (Listed in

methods section I) for 1 hours, and then exposed to bacteria, toxins or cytotoxic reagents.

Cell Lines. A549 cells were grown in DMEM, 10% FBS, at 37°C, 5% CO_2. Normal Human Bronchial Epithelial Cells (NHBEC) were grown in bronchial epithelial cell basal medium supplemented with bronchial epithelial growth medium single quots (Lonza, Allendale, NJ). All cells were incubated at 37°C, 5% CO_2. Cells were plated at a final concentration of 5 x 10^4 cells/well in 96-well plates and incubated at 37°C, 5% CO_2.

Pneumolysin neutralization experiments. Cell cytotoxicity of pneumolysin in A549 cells was neutralized using PLY-4 (5) (# ab71810, Abcam) at 100ng. To test the immunogenic properties of the necroptosis associated with caspase activation, A549 cells were pre-treated with Z-VAD-FMK, or mock treated, then cells were challenged with pneumolysin for 4 hours. Cell supernatant was treated with pneumolysin neutralizing antibody, PLY-4 at a concentration of 100ng, then used to treat inactive THP-1 human macrophages. Untreated IL-6 control shows release of IL-6 after challenge with the supernatant of untreated/unchallenged A549 cells. The conditioned media for each experimental condition was checked for IL-6 and the obtained values were subtracted from the THP-1 obtained values.

Immunoblots. Whole-cell extracts were prepared with RIPA buffer (150mM NaCl, 1% NP-40, 0.5% DOC, 0.1% SDS and 50mM Tris HCl) containing protease/phosphatase inhibitors (Pierce, Thermo Scientific, Waltham, MA, USA). The samples were run on TGX Stain-free polyacrylamide gels (Bio-Rad) by SDS-PAGE, under reducing conditions. Total protein was visualized before transfer on stain-free gels on a ChemiDoc XRS+ (Bio-Rad). Proteins were transferred onto nitrocellulose using a Trans-Blot Turbo Transfer System (Bio-Rad). Western blots were performed using standard methods and visualized using a Bio-Rad ChemiDoc XRS+ and Image Lab Software. The proteins from human cells were detected with antibodies against MLKL (1:1000, ab184718, Abcam), pMLKL (1:1000, 91689, Cell Signaling Technology), caspase-1 (1:500, orb216042, Biorbyt), cleaved-caspse-1 (1:1000, ab179515, Abcam), caspase-2 (2µg/ml, GTX59706, GeneTex), cleaved caspase-2 (1:500, GTX866954, GeneTex), caspase-3 (2µg/ml,559565, BD Pharmingen) and cleaved caspase-3 (1:500, 9664S, Cell Signaling Technology), caspase-4 (1:200, sc-56056, Santa Cruz), cleaved caspase-4 (1:1000, 11856-1-AP, ProteinTech), caspase-8 (1µg/ml, ab25901, Abcam), caspase-10 (2µg/ml, GTX59704, GeneTex), and cleaved caspase-10 (1:200, sc-22184, Santa Cruz). The mouse samples were detected with antibodies against MLKL (1:1000, ab172868, Abcam), cleaved caspase-2 (1:500, GTX866954, GeneTex), caspase-11/casp-4 (1:1000, MAB8648, R&D Systems), and pMLKL (1:1000, ab196436, Abcam). Corresponding secondary antibody, horseradish peroxidase conjugated goat anti-rabbit or anti-mouse IgG, was used at 1:10,000 dilution (Jackson ImmunoResearch, West Grove, PA, USA). To confirm protein load, membranes were also probed with antibody against cytoskeletal actin at 1:5,000 (Bethyl Laboratories Inc., Montgomery, TX)

Fluorescent-labeled inhibitor of caspases/ Propidium Iodide Staining. Propidium iodide (PI) and fluorescent-labeled inhibitors of caspases (FLICA) (ImmunoChemistry,

Bloomington, MN) were used to stain DNA in cells that have lost membrane integrity (i.e. necrotic cells) and activation of caspases in A549 lung epithelial cells, respectively. According with the manufacturer instructions, 2 hours following infection with *Sma,* challenged with rPly or α-toxin wells were washed with the supernatant set aside. Specific FLICA was added to the cells diluted 1:30 and incubated for 30-60 minutes at 37°C. Cells were then washed and spun at 200xg for 5 minutes. PI was then added at a concentration of 0.5% v/v and incubated for 5 minutes at room temperature. DAPI was added to the cells for 1 minute at room temperature. Cells were analyzed using fluorescent microscopy.

Immunofluorescent Antibodies. Labeling of pMLKL (Alexa Fluor 647), cleaved caspase-2, -4 and -10 (Alexa Fluor 488) antibodies was executed with Alexa Fluor Antibody Labeling kits (Life Technologies Thermo Fisher, Waltham, MA) following manufacturer instructions. Clathrin antibody was obtained from Santa Cruz (sc-6579, Dallas, TX).

Enzyme-Linked Immunosorbent Assay. IL-6 ELISA (Bio-Techne Corporation, Minneapolis, MN), KC and IL-1α (BD Biosciences, MD, USA) were used following the manufacturer instructions.

References:

1. Gonzalez-Juarbe N, Mares CA, Hinojosa CA, Medina JL, Cantwell A, Dube PH, et al. Requirement for Serratia marcescens cytolysin in a murine model of hemorrhagic pneumonia. Infection and immunity. 2015;83(2):614-24.
2. Brown AO, Mann B, Gao G, Hankins JS, Humann J, Giardina J, et al. Streptococcus pneumoniae translocates into the myocardium and forms unique microlesions that disrupt cardiac function. PLoS pathogens. 2014;10(9):e1004383.
3. Gonzalez-Juarbe N, Bradley KM, Shenoy AT, Gilley RP, Reyes LF, Hinojosa CA, et al. Pore-forming toxin-mediated ion dysregulation leads to death receptor-independent necroptosis of lung epithelial cells during bacterial pneumonia. Cell Death Differ. 2017.
4. Gonzalez-Juarbe N, Gilley RP, Hinojosa CA, Bradley KM, Kamei A, Gao G, et al. Pore-Forming Toxins Induce Macrophage Necroptosis during Acute Bacterial Pneumonia. PLoS pathogens. 2015;11(12):e1005337.
5. de los Toyos JR, Mendez FJ, Aparicio JF, Vazquez F, Del Mar Garcia Suarez M, Fleites A, et al. Functional analysis of pneumolysin by use of monoclonal antibodies. Infection and immunity. 1996;64(2):480-4.
